# Effect of Covalent Organic Frameworks Containing Different Groups on Properties of Sulfonated Poly(ether ether ketone) Matrix Proton Exchange Membranes

**DOI:** 10.3390/nano12193518

**Published:** 2022-10-08

**Authors:** Xiaoyu Meng, Yinan Lv, Lei Ding, Luman Peng, Qiwang Peng, Chuanbo Cong, Haimu Ye, Qiong Zhou

**Affiliations:** 1 Department of Materials Science and Engineering, College of New Energy and Materials, China University of Petroleum, Beijing 102249, China; 2 Beijing Key Laboratory of Failure, Corrosion, and Protection of Oil/Gas Facilities, China University of Petroleum, Beijing 102249, China; 3 CSSC Systems Engineering Research Institute, Beijing 100036, China

**Keywords:** PEMs, SPEEK, proton conductivities, dimensional stabilities, covalent organic frameworks

## Abstract

The rich −SO_3_H groups enable sulfonated poly (ether ether ketone) (SPEEK) to possess excellent proton conductivities in proton exchange membrane (PEM), but cause excessive water absorption, resulting in the decline of dimensional stability. It is a challenge to resolve the conflict between conductivity and stability. Owing to its unique structural designability, covalent organic frameworks (COFs) have been used to regulate the performances of PEMs. The authors propose the use of COFs with acidic and basic groups for meeting the requirements of proton conductivity and dimensional stability. Herein, COFs containing different groups (sulfoacid, pyridine, and both) were uniformly dispersed into the SPEEK matrix by in situ synthesis, and the effects on the properties of SPEEK matrix PEMs were revealed. The sulfoacid group significantly improves proton conductivities. At 60 °C, under 95% RH, the conductivity of the SPEEK/TpPa−SO_3_H-20 composite membrane was 443.6 mS·cm^−1^, which was 3.3 times that of the pristine SPEEK membrane. The pyridine group reduced the swelling ratio at 50 °C from 220.7% to 2.4%, indicating an enhancement in dimensional stability. Combining the benefits of sulfoacid and pyridine groups, SPEEK/TpPa−(SO_3_H-Py) composite membrane has a conductivity of 360.3 mS·cm^−1^ at 60 °C and 95% RH, which is 1.86 times that of SPEEK, and its swelling ratio is 11.8%, about 1/20 of that of SPEEK membrane. The method of in situ combination and regulation of groups open up a way for the development of SPEEK/COFs composite PEMs.

## 1. Introduction

Proton exchange membrane fuel cells (PEMFCs) have the advantages of high energy density, low operating temperature, fast start-up speed, and simple structure [1,2,3]. Playing an important role in PEMFCs, proton exchange membranes (PEMs) need to have high proton conductivity, excellent thermal stability, and mechanical stability [4]. As the most widely used commercially available PEMs, Nafion is a fluoropolymer made of sulfonated polytetrafluoroethylene, which has high proton conductivities [5]. However, problems such as high cost, complex preparation process, and reduced dimensional stabilities, proton conductivities in high-temperature environments, and unstable relative humidity (RH) limit the further application of Nafion [5,6]. Therefore, it has become a research hotspot to find alternative materials for PEMs with low prices and great properties.

Sulfonated polyether ether ketone (SPEEK), as a potential PEM material, has excellent mechanical properties, and a chemical stability [7]. Similar to the Nafion microphase structure, SPEEK is composed of hydrophilic domains and hydrophobic domains. As the hydrophobic domains, the PEEK skeleton improves mechanical properties [4]. The hydrophilic domains of SPEEK are composed of −SO_3_H side groups, which play the role of proton conductors. The hydrophilic domains (−SO_3_H and water) form spherical ion clusters. As the water content increases, the number of spherical ion clusters increases, and these spherical ion clusters connect to form connected proton transport channels [8,9,10,11]. SPEEK with a high degree of sulfonation (DS) is more likely to form ion clusters and has higher proton conductivities [12]. However, with it comes swelling caused by excessive water absorption, especially at higher service temperatures. This reduces the dimensional stability of the PEMs and also limits the operating temperature range of SPEEK-based PEMs. To overcome this shortcoming, some second phases are added to the SPEEK matrix, such as graphene oxide (GO), metal–organic frameworks (MOFs), and covalent organic frameworks (COFs). More active groups can be introduced into SPEEK ionomers as proton conductors to improve the connectivity of ion clusters, reduce the activation energy required for proton transport, and effectively improve proton conductivity. Therefore, a stable and effective carrier is needed to introduce active groups into the SPEEK matrix.

More second-phase particle-doped polymer PEMs have been reported. If there is no ideal compatibility between the doped particles and the polymer matrix, the phase separation phenomenon will occur, which will make work unsatisfactory. The excellent compatibility between covalent organic frameworks (COFs) and polymer matrices makes them have great potential for application in PEMs [13]. COFs are a type of nanoparticles that connect organic monomers through strong covalent bonds and are formed by orderly stacking by non-covalent interactions. Compared with the traditional crystalline porous structure, COFs can accurately pre-design the topological structure and design functional COFs to realize the structure and chemical control of specific functions [14,15,16]. In addition, diverse structural units, stable porosity, and thermal stability enable COFs to be used in gas storage and separation [17], catalysis [18], sensing [19], photoelectric [20], and proton conduction [21]. Using molecular design and structural optimization, the content, and location of acidic groups and basic groups in COFs materials can be precisely controlled. Peng et al. [22] successfully prepared fully sulfonated COFs (NUS−10) by reacting amino monomers containing sulfonate groups with Tp. It was found that the proton conductivity of TpPa−1 (which had the same structure but does not contain sulfonic acid groups) at room temperature and 97% RH was only 2.40 × 10^−5^ S·cm^−1^, which was almost negligible. The proton conductivity of NUS−10 was as high as 3.96 × 10^−2^ S·cm^−1^ under the same conditions. Adding acidic groups in the confined nanospace can effectively increase the proton conductivity of COFs. The participation of basic groups will allow protons to be transported along the hydrogen bond network composed of acids and bases, thereby improving the efficiency of proton transfer [23]. TpPa−(SO_3_H-Py) containing both sulfonic acid groups and pyridine groups were successfully synthesized, in which the sulfonic acid groups were used as the acidic sites and the pyridyl groups were used as the basic sites, and ideal proton conductivities were obtained [24].

Based on the unique molecular structure of the porous material COFs, it provides more options for improving the performance of PEMs. COFs exist in the form of a filler in polymer, which can not only form acid–base interactions with the polymer to obtain a high proton conductive electrolyte material but also inhibit the swelling behavior of the polymer in solution. However, COFs are usually presented in the form of fluffy microcrystalline powder, with a hard texture and low solubility, making it difficult to post-process COFs powder. Therefore, how to uniformly disperse COFs powder into a polymer matrix is an urgent problem to be solved. To improve the compatibility of COFs and polymer matrix, Yin et al. [25] chose a Schiff base COFs network (SNW−1) with inherent amino groups and introduced it into the Nafion matrix to prepare composite membranes. The −N−H− bond in the SNW−1 structure can interact with −SO_3_H in Nafion to improve the dispersion of SNW−1 in the matrix. Fan et al. [26] doped triazole functionalized COFs into SPEEK matrix and used triazole groups and −SO_3_H groups to form acid-base pairs at the interface to obtain uniformly dispersed composite membranes. The proton conductivity of the SPEEK/HPW@COF composite membrane at 65 °C and 40% RH was 35.5 times that of SPEEK. In general, these methods have obvious limitations on the choice of COFs.

Herein, we improved the dispersion of COFs in the SPEEK matrix by in situ synthesis, thereby improving the proton conductivity and mechanical properties of PEMs. At the same time, the performances of the PEMs were further improved by adjusting the COFs’ frame structure. We prepared TpPa−SO_3_H and TpPa−Py by the in situ synthesis in the SPEEK matrix, respectively. TpPa−SO_3_H can provide additional proton conduction sites for proton conduction and improve the proton conductivity of PEMs. The pyridine group of TpPa−Py had a strong interaction with the −SO_3_H group of the SPEEK matrix and improved the mechanical stability of PEMs. The −SO_3_H and pyridine groups had excellent performance in improving the proton conductivity and mechanical stability of PEMs, respectively. Therefore, the −SO_3_H and pyridine groups were introduced into the COFs structure at the same time to improve the comprehensive performance of the PEMs.

## 2. Materials and Methods

### 2.1. Materials and Chemicals

Poly (ether ether ketone) (VICTREX^®^ 450 PF) was produced by Victrex (Lancashire, England); concentrated H_2_SO_4_ was used as a sulfonating agent to achieve SPEEK as described in previous work [27]. 1,3,5-triformylphloroglucinol (Tp), 2,5-diaminobenzene-sulfonic acid (Pa−SO_3_H), 2,5-diaminopyridine (Pa−Py) were purchased from Sigma-Aldrich Co., Ltd. (Shanghai, China). All the other reagents mentioned in this work were produced by Beijing chemical reagent factory (Beijing, China).

### 2.2. Synthesis of SPEEK

PEEK powder (25 g) was added into concentrated H_2_SO_4_ (250 mL), which was stirred at 50 °C. After PEEK was completely dissolved into H_2_SO_4_, the solution was vigorously stirred for another 4 h 10 min. The reaction was terminated by pouring the sulfonated polymer solution into ice water under mechanical stirring. The precipitated SPEEK was repeatedly washed using deionized (DI) water until the pH of the rinse water was approximately 7. The SPEEK material was obtained after stirring again for 2 h and drying in a blast oven at 60 °C for 48–72 h.

### 2.3. Preparation of SPEEK/COF Composite Membranes

The SPEEK was added to DMSO, stirred at 60 °C until SPEEK was dissolved, and formed a SPEEK/DMSO solution with a mass concentration of 20%. A 20 wt% SPEEK/DMSO (4.66 mL) solution was cast in a glass mold and kept in an oven under 80 °C for 72 h to prepare the SPEEK membrane. Pa−SO_3_H and PTSA were placed in a mortar and ground for 5–10 min to form a uniform mixed powder. Tp was added to the above-mixed powder and grinding continued for 5 min. Then, deionized water (150 μL) was added, and ground for 20 min to make a uniform slurry. SPEEK/DMSO solution was added to the above slurry and ground for about 30 min to make it uniformly mixed to obtain a film-forming liquid. The proportion of each raw material is shown in the supporting information. The film-forming solution was coated on a glass plate and dried in a blast drying oven at 80 °C for 72 h to obtain an in situ synthesized SPEEK/TpPa−SO_3_H composite PEM.

**Scheme 1:**
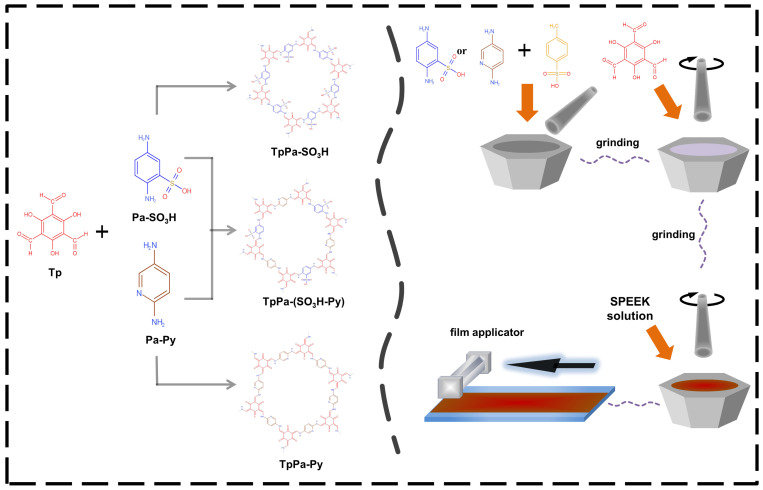
Synthetic route of SPEEK/COFs composite membranes.

The synthesis process of TpPa−Py and TpPa−(SO_3_H-Py) is the same as the synthesis process of TpPa−SO_3_H, and the usage ratio of each raw material is shown in Tables S1–S3. The monomers and process diagram used to prepare composite membranes are shown in Scheme 1.

### 2.4. Characterizations

X-ray diffraction (XRD, Bruker D8Focus, Bremen, Germany) was utilized to analyze the physical topology crystallization of COF nanoparticles. The chemical composition of COF nanoparticles was characterized by Fourier transform infrared (FTIR, Bruker Tensor II, Bremen, Germany). The thermal stability of SPEEK and SPEEK/COF membranes were evaluated through thermal gravimetric analysis under the nitrogen atmosphere in the range of 40 °C to 800 °C (TGA, TA Q5000, New Castle, DE, USA) (Figure S1). The scanning electron microscope (SEM, HITACHI SU8010, Tokyo, Japan) and transmission electron microscopy (TEM, FEI F20, Oregon, USA) were utilized to characterize the COF feature and the cross-section morphologies of as-prepared membranes. Solid-state nuclear magnetic resonance (^13^C SSNMR, JEOL JNM-ECZ600R, Japan) was used to characterize the structure of COF. The mechanical properties of the composite membranes were measured on a universal material testing machine (KQL, WDL-10, Yangzhou, China) at a strain rate of 10 mm/min.

### 2.5. Water Uptaking and Swelling Ratio

The dry membranes were cut into rectangle samples (about 1 cm × 3 cm) and its mass (*W_dry_*) and area (*S_dry_*) were recorded. Then, the samples were immersed in deionized water at different temperatures for 12 h, and the moisture on the surface of the membrane was quickly removed, and then the wet weight (*W_wet_*) and wet area (*S_wet_*) of the samples were measured. Each sample was measured at least three times and the average value was calculated. The water absorption and swelling degree of the film is calculated by the following formula:

(1) $$\mathrm{Water}\;\mathrm{Uptaking}\;=\frac{\left(W_{wet}-W_{dry}\right)}{\left(W_{dry}\right)}\times 100\%$$

(2) $$\mathrm{Area}\;\mathrm{swelling}\;\mathrm{ratio}\;=\frac{\left(S_{wet}-S_{dry}\right)}{\left(S_{dry}\right)}\times 100\%$$

### 2.6. Proton Conductivity

The proton conductivity was characterized by CHI660E. The test was carried out under different temperature and relative humidity (RH) conditions in the YSGTS-50 constant temperature and humidity test chamber. All membrane samples were cut into 1.5 cm × 3 cm sample strips before the test and placed in a constant temperature and humidity test chamber to be sufficiently stable under the tested conditions. The proton conductivity of the membrane was calculated according to Equation (3):

(3) $$\sigma =\frac{L}{AR}\;$$

where *σ* represents the proton conductivity of the membrane (mS·cm^−1^), *L* is the distance between the two platinum electrodes (cm), *R* (Ω) is the resistance value of the membrane, and A is the cross-sectional area of samples.

### 2.7. Ion Exchange Capacity (IEC) and Degree of Sulfonation (DS)

The ion exchange capacity (IEC) was determined by trans titration method. The membranes were soaked in 2 mol·L^−1^ NaCl solution for 24 h, the purpose was to completely replace the H^+^ in the –SO_3_H groups of membranes with Na^+^ in the NaCl solution. Phenolphthalein solution was used as indicator, and the replaced acidic NaCl solutions were titrated with 0.01 mol·L^−1^ NaOH solution. The calculation formula of IEC is according to Equation (4):

(4) $$IEC=\frac{C_{NaOH}\times V_{NaOH}}{M_{dry}}$$

where *V_NaOH_* is the volume of NaOH solution used for titration; *C_NaOH_* is the concentration of NaOH solution; *M_dry_* is the mass of dry samples of membranes used for measurement.

The degree of sulfonation (DS) of the SPEEK pristine membrane can be calculated from the theoretical value of IEC with the following formula:

(5) $$\mathrm{DS}\;=\frac{288\cdot IEC}{1000-102\cdot IEC}\times 100\%$$

## 3. Results and Discussion

### 3.1. SPEEK/TpPa−SO_3_H and SPEEK/TpPa−Py Composite Membranes

#### 3.1.1. Characterization of TpPa−SO_3_H and TpPa−Py

**Figure 1:**
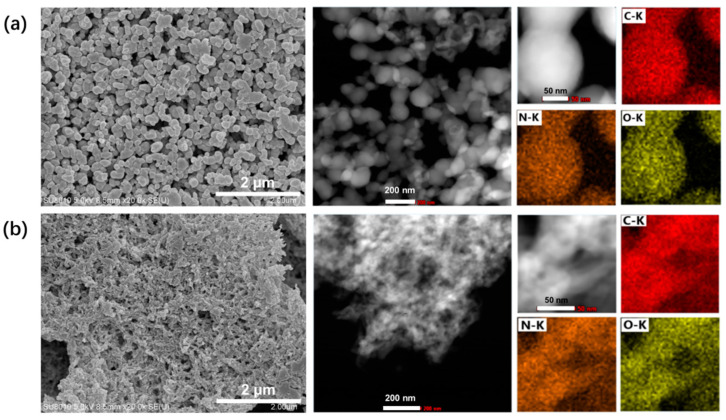
SEM, TEM and EDS-mapping of (**a**) TpPa−SO_3_H and (**b**) TpPa−Py.

TpPa−SO_3_H and TpPa−Py were selected to be to be synthesized in situ in SPEEK matrix to prepare PEMs. To characterize the synthesis of TpPa−SO_3_H and TpPa−Py in the membranes, the composite membranes were dissolved several times in DMSO solvent at 80 °C and centrifuged, and then the COFs powders were separated from it. The morphology of TpPa−SO_3_H and TpPa−Py were obtained by SEM and TEM (Figure 1). Consistent with previously reported in the literature [24,28], the morphology of TpPa−SO_3_H was spherical, and the diameter of each spherical particle is about 100 nm. The morphology of TpPa−Py was fibrous, which is also nanoscale in size. Compared with the SPEEK matrix, the characteristic element of COF is N. It can be seen from the EDS results that the washed powder contained the N element, and the distribution of the N element in the powder was uniform.

**Figure 2:**
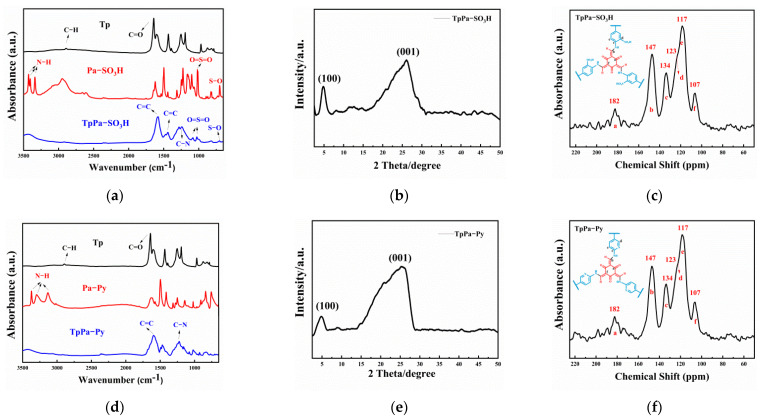
(**a**) FTIR, (**b**) XRD, (**c**) NMR spectra of TpPa−SO_3_H and (**d**) FTIR, (**e**) XRD, (**f**) NMR spectra of TpPa−Py.

The FTIR spectrum (Figure 2a,d) confirmed the successful synthesis of TpPa−SO_3_H and TpPa−Py in SPEEK matrix. The complete consumption of the raw materials was indicated by the absence of the characteristic N−H stretching band (3428–3338 cm^−1^) of the free diamine and aldehydic –CH=O (1643 cm^−1^) of the free Tp. The absorption band at 2893 cm^−1^ corresponded to the C−H stretching peak of the Tp aldehyde group [29]. The nearby peaks at 1590 cm^−1^ and 1230 cm^−1^ were attributed to the stretching vibration peaks of C=C and C−N, which proved the enol-ketone mutual variation structured [30]. The peaks at 1084 cm^−1^ and 1027 cm^−1^ were attributed to the stretching vibration peaks of sulfonate O=S=O on TpPa−SO_3_H [31].

The ordered structure of TpPa−SO_3_H and TpPa−Py was determined by XRD (Figure 2b,e). There was a diffraction peak at 2θ = 4.9°, corresponding to the reflection of the (100) plane of the TpPa−SO_3_H lattice [32]. Due to the reflection from the (001) plane, a broad peak appeared near 26°, indicating that there was a π–π stacking between COF single molecules [33]. The diffraction peak at 4.9° corresponded to the reflection peak of the TpPa−Py (100) plane. The broad peak at 26.0º at the (001) plane confirmed that TpPa−Py was formed in the form of crystals and π–π stacks [24].

To analyze the structure of the TpPa−SO_3_H and TpPa−Py samples separated from composite membranes, a ^13^C SSNMR test was performed (Figure 2c,f). The carbonyl carbon peak of TpPa−SO_3_H and TpPa−Py at 182 ppm indicated the presence of typical keto-enol tautomers. The signals near 123 ppm and 117 ppm correspond to carbon atoms near the sulfonic acid group in TpPa−SO_3_H and the pyridine group in TpPa−Py. Moreover, the peak intensity at 117 ppm is significantly higher than other peaks, which indicated that the carbon in the residual SPEEK in the sample also participates in the resonance. All the results of SEM, TEM, FTIR, XRD, and ^13^C SSNMR showed that TpPa−SO_3_H and TpPa−Py were successfully synthesized in the composite membranes.

#### 3.1.2. Proton Conductivities of SPEEK/TpPa−SO_3_H and SPEEK/TpPa−Py Composite Membranes

**Figure 3:**
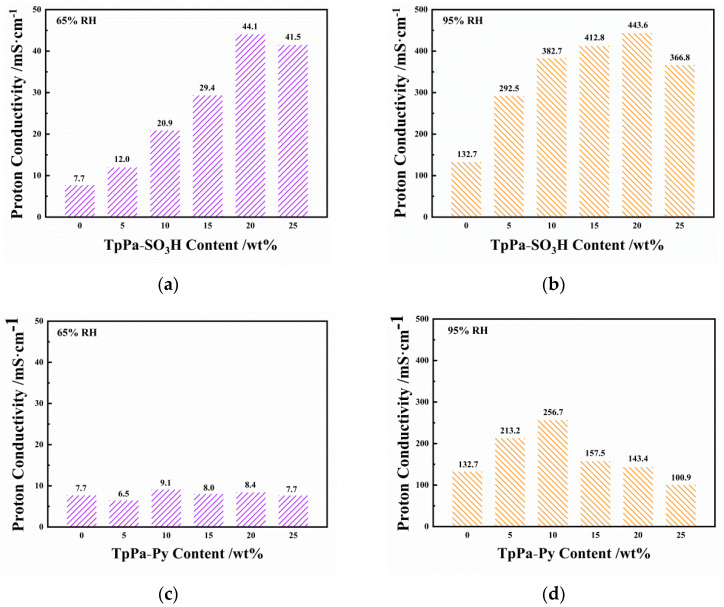
Proton conductivity at 60 °C, (**a**,**c**) 65% RH and (**b**,**d**) 95% RH of SPEEK, SPEEK/TpPa−SO_3_H and SPEEK/TpPa−Py.

Proton conductivity is an important factor in evaluating the performance of proton exchange membranes. The proton conductivity of SPEEK, SPEEK/TpPa−SO_3_H, and SPEEK/TpPa−Py composite membranes at 60 °C, under 65% RH and 95% RH was shown in Figure 3, respectively. After adding TpPa−SO_3_H and TpPa−Py nanoparticles, the proton conductivity of the SPEEK/COF membranes continued to increase with the addition of COF nanoparticles and reached the maximum value at 20 wt% and 10 wt%, respectively. Under the humidity of 65% RH and 95%, the satisfactory proton conductivity exhibited by the SPEEK/TpPa−SO_3_H-20 composite membrane which was 44.1 mS·cm^−1^ and 443.6 mS·cm^−1^, 5.7 times and 3.3 times than SPEEK membranes, respectively. Meanwhile, the higher proton conductivity exhibited by the SPEEK/TpPa−Py-10 composite membrane was 9.1 mS·cm^−1^ and 256.7 mS·cm^−1^, 1.2 times and 1.9 times of SPEEK membranes, respectively. The decline of proton conductivity appeared at the addition content of 25 wt% TpPa−SO_3_H and 15 wt% TpPa−Py. TpPa−SO_3_H and TpPa−Py agglomerated in the SPEEK matrix making it difficult to form a connected proton channel in the membrane. Protons are mainly transferred through water or acidic sites. The framework of TpPa−SO_3_H contains both the acidic group −SO_3_H and the basic group −C−N−, which can improve the water retention of the composite membrane under low humidity and accelerate the dissociation of the proton donor (−SO_3_H). Moreover, the good dispersion of TpPa−SO_3_H provided ordered sulfonic acid groups, which increased the efficiency of proton conduction, and promoted the rapid transmission of protons. Therefore, the SPEEK/TpPa−SO_3_H composite membranes exhibited excellent proton conductivity in both low humidity and high humidity. Compared with the acidic sulfonic acid group, the basic pyridine group in TpPa−Py has weaker hydrophilicity. However, the pyridine group and the sulfonic acid group can form acid–base pairs to improve the efficiency of proton transfer. The above reasons make SPEEK/TpPa−SO_3_H and SPEEK/TpPa−Py membranes had considerable proton conductivity in high- and low-humidity environments. Compared with TpPa−Py, TpPa−SO_3_H had a more obvious effect on improving the proton conductivity.

After testing, it can be seen that the DS of the SPEEK used in the experiment was about 47.3%. According to Table 1, it can be seen that the IEC change trend of the SPEEK/TpPa−SO_3_H composite membranes is consistent with the proton conductivities. The IEC increased with the increase in the TpPa−SO_3_H content. When the TpPa−SO_3_H content is 20 wt%, the IEC reached 2.26 mmol·g^−1^, and the IEC reached the highest of the SPEEK/TpPa−SO_3_H composite membranes. When the amount of TpPa−SO_3_H content increased to 25 wt%, the IEC decreased slightly. The IEC of the SPEEK/TpPa−Py composite membranes is generally lower than that of the SPEEK/TpPa−SO_3_H composite membranes, reaching 1.61 mmol·g^−1^ when the TpPa−Py content was 10 wt%, which was the highest value in the SPEEK/TpPa−Py composite membranes.

#### 3.1.3. Dimensional Stability

**Figure 4:**
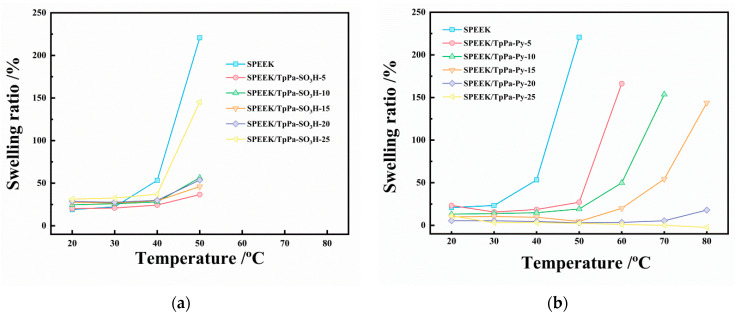
Swelling ratio of (**a**) SPEEK and SPEEK/TpPa−SO_3_H, (**b**) SPEEK and SPEEK/TpPa−Py at different temperatures under 100% RH.

Swelling is an important parameter to characterize the performance of PEM. Water uptaking is closely related to the free volume and hydrophilicity of the membrane and plays an important role in the transport of protons (Figure S3a,b). However, excessive swelling caused by excessive water uptaking may destroy the chemical stability of the membrane. The swelling ratios of the composite membranes at 20 °C–50 °C were shown in Figure 4. The swelling ratio of the SPEEK membrane at 50 °C was as high as 220.7%. While the swelling ratio of SPEEK/TpPa−SO_3_H-20 at 50 °C was 53.7%, which was only about 1/4 of SPEEK. Owing to the hydrophilic groups −SO_3_H attached to the TpPa−SO_3_H skeleton, the SPEEK/TpPa−SO_3_H composite membrane can combine with more water molecules at low temperatures, which improves the proton conductivity of the composite membrane, but the swelling ratio of the composite membrane will also increase with the increase in temperature. Due to the interaction such as the hydrogen bond between TpPa−SO_3_H and the polymer chain, which limits the migration of the polymer chain, the addition of TpPa−SO_3_H to the SPEEK matrix can significantly inhibit the swelling of the composite membranes, especially at high temperatures. Compared with TpPa−SO_3_H, TpPa−Py had a greater effect on improving the dimensional stability of PEM. The swelling ratio of SPEEK/TpPa−Py composite membranes was lower than that of SPEEK membranes. In the hydrated state at 50 °C, the area swelling ratio of SPEEK/TpPa−Py series membranes was in the range of 3–27.1%. With the content of TpPa−Py increased, the swelling ratio of the composite membranes decreased. When the temperature was higher than 50 °C, membranes absorbed water and swelled excessively, and the sizes of composite membranes cannot be measured. However, the composite membranes of SPEEK/TpPa−Py-20 and SPEEK/TpPa−Py-25 can still maintain excellent dimensional stability at 80 °C. The excellent inhibitory effect of TpPa−Py on the swelling ratio of the composite membranes is mainly due to the pyridine group in the TpPa−Py skeleton forming an ionic cross-linking network in the polymer matrix, which can effectively limit the excessive expansion of the SPEEK matrix.

#### 3.1.4. Structure and Morphology Characterization of SPEEK/TpPa−SO_3_H and SPEEK/TpPa−Py Composite Membranes

**Figure 5:**
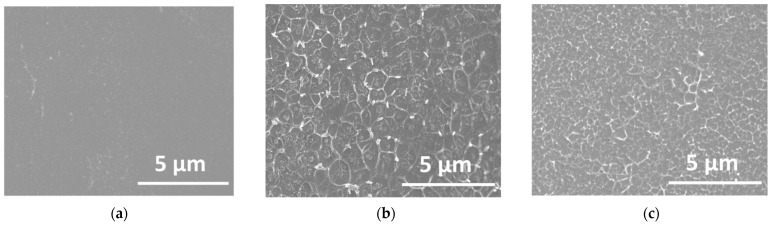
SEM cross-section images of (**a**) SPEEK, (**b**) SPEEK/TpPa−SO_3_H-15, (**c**) SPEEK/TpPa−Py-15.

The cross-sectional scanning electron microscopy images of SPEEK, SPEEK/TpPa−SO_3_H-15, and SPEEK/TpPa−Py-15 composite membranes were shown in Figure 5. Compared with SPEEK, the cross sections of the composite membranes were rougher, but the textures were uniform, indicating that COFs were evenly dispersed in the matrix. Figure S2a–k showed the cross-sectional scanning electron microscopy images of all the SPEEK, SPEEK/TpPa−SO_3_H, and SPEEK/TpPa−Py composite membranes. With the increase in the amount of TpPa−SO_3_H and TpPa−Py nanoparticles, the composite membranes maintained a compact structure. Prepared by the in situ composite method, the polymer chains of SPEEK hindered the π–π interaction between COF particles, [34] and the −NH− in the COF framework can interact with the −SO_3_H in the SPEEK matrix to improve compatibility between COF and SPEEK matrix. Moreover, the pyridine group and secondary amine group in the TpPa−Py structure had a strong interaction with the sulfonic acid group of SPEEK, which reduced the agglomeration of TpPa−Py in the matrix. The interaction between the COF and SPEEK matrix was beneficial to maintain the mechanical properties of composite membranes.

#### 3.1.5. Mechanical Properties

**Figure 6:**
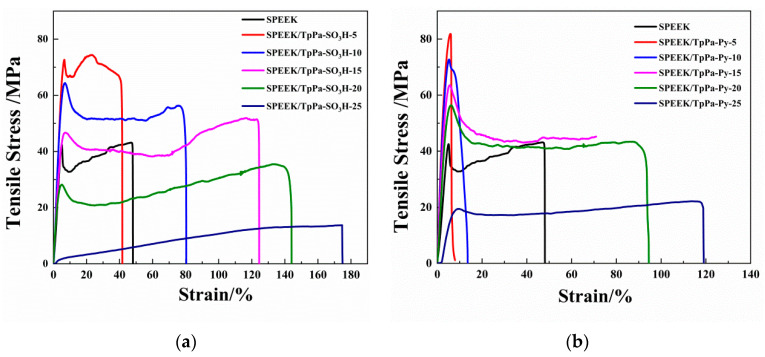
Stress–strain curves of (**a**) SPEEK and SPEEK/TpPa−SO_3_H, and (**b**) SPEEK and SPEEK/TpPa−Py.

Figure 6 showed the stress–strain curves of SPEEK, SPEEK/TpPa−SO_3_H (Figure 6a), and SPEEK/TpPa−Py (Figure 6b) composite membranes at 25 °C. The hydrophobic backbone in SPEEK polymer chain makes membranes have good mechanical properties. The SPEEK membrane showed tensile stress of 42.5 MPa. When the loading of TpPa−SO_3_H was 5 wt%, the tensile strength of the composite membranes was as high as 47.6 MPa, indicating that the introduction of TpPa−SO_3_H improved the tensile strength of the composite membranes. There were two reasons for the improvement in mechanical properties. First, the ketoenamine (−C−N−) on the TpPa−SO_3_H ring can interact with the sulfonic acid and (−SO_3_H) acid base on the SPEEK aromatic ring. At the same time, π–π interaction may also occur between the TpPa−SO_3_H framework and the SPEEK benzene ring [26]. The curve shows that the tensile strength of the composite film decreases with the increase in TpPa−SO_3_H filling amount because the content of PTSA in the TpPa−SO_3_H channel increases with the increase in the content of TpPa−SO_3_H. As a small molecule, PTSA increases the separation of polymer chains and reduces the intermolecular force of polymers [35]. When the filling amount of TpPa−SO_3_H is 25 wt%, it will inevitably agglomerate in the membrane, and plasticization of PTSA led to an increase in elongation. As shown in Figure 6b, the tensile strength of all SPEEK/TpPa−Py composite membranes was higher than that of the SPEEK membrane. When the filling amount of TpPa−Py was 5 wt%, the tensile strength of the composite membranes was as high as 81.8 MPa. The hydrogen bond interaction between the pyridine group in the TpPa−Py and the sulfonic acid group in SPEEK increased the yield strength of the composite membranes [36]. To a certain extent, this strong interaction can inhibit polymer chain migration.

### 3.2. SPEEK/TpPa−(SO_3_H-Py) Composite Membrane

#### 3.2.1. Characterization of SPEEK/TpPa−(SO_3_H-Py)

**Figure 7:**
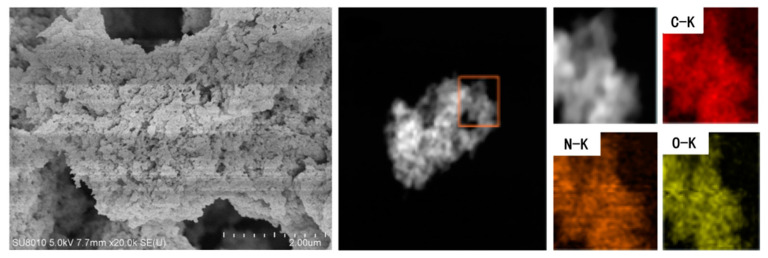
SEM, TEM, and EDS-mapping of TpPa−(SO_3_H–Py).

From the above results, it is found that the −SO_3_H group has an excellent performance in promoting proton conduction and the pyridine group can effectively improve the dimensional stability and strength of PEMs. Therefore, the −SO_3_H group and the pyridine group were simultaneously introduced into the COF structure to improve the performances of PEMs. The content of TpPa−(SO_3_H-Py) was 20 wt% and the ratio of Pa−SO_3_H to Pa−Py was 1:1. Therefore, TpPa−(SO_3_H-Py) was synthesized and added to the SPEEK matrix by in situ synthesis to prepare composite membranes. To characterize the synthesis of TpPa−(SO_3_H-Py) in the membrane, the composite membranes were dissolved in DMSO at 80 °C, and then TpPa−(SO_3_H-Py) was separated from the solution. The morphology of TpPa−(SO_3_H-Py) was obtained by SEM and TEM (Figure 7). Consistent with the previously reported literature [24], the morphology of (TpPa−(SO_3_H-Py)) was not a simple mixture of spherical and fibrous, but formed by stacking layers on each other. Due to the centrifugal force, the TpPa−(SO_3_H-Py) is stacked together, but it can still be seen that the TpPa−(SO_3_HPy) is also composed of nanoparticles (please see Figure S4 for other SEM images of TpPa−(SO_3_H-Py)). Compared with SPEEK matrix, the characteristic element of COF is N. It can be seen from the EDS of the washed powder that the washed powder contained the N element, and the distribution of the N element in the powder was uniform.

**Figure 8:**
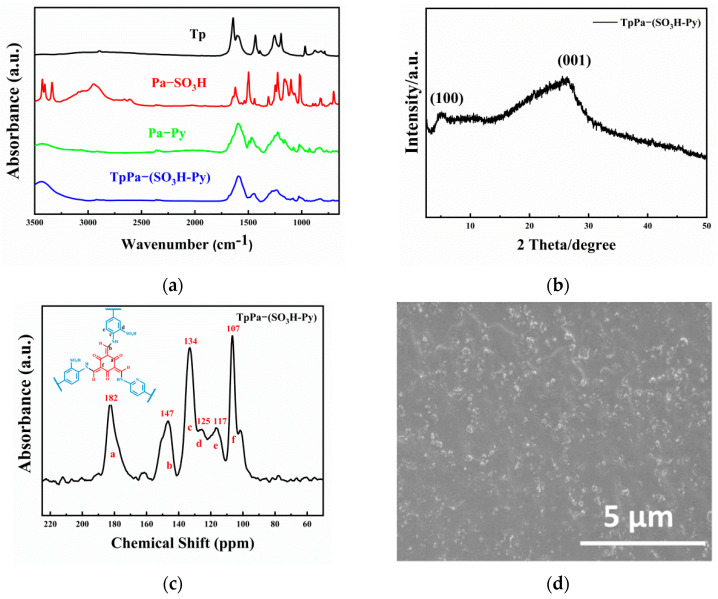
(**a**) FTIR, (**b**) XRD, (**c**) NMR spectra of TpPa−(SO_3_H-Py), (**d**) SEM cross-section images of SPEEK/TpPa−(SO_3_H-Py).

Figure 8a showed the FTIR spectra of TpPa−(SO_3_H-Py). The TpPa−(SO_3_H-Py) sample had characteristic peaks of C=C (1583 cm^−1^) and C−N (1232 cm^−1^), which proved the existence of enol-ketone mutual variation structure. The peaks at the wavenumbers of 1084 cm^−1^ and 1027 cm^−1^ were attributed to the O=S=O stretching vibration peaks of TpPa−(SO_3_H-Py). The ordered structures of TpPa−(SO_3_H-Py) were characterized by XRD (Figure 8b). Corresponding to the reflection of the (100) plane, there was a peak at 4.9° in the TpPa−(SO_3_H-Py) sample spectrum. The peak at 26° corresponded to the reflection peak of the (001) plane, which proved the existence of π–π stacking in the synthesis process of TpPa−(SO_3_H-Py). Figure 8c showed the ^13^C SSNMR spectrum of TpPa−(SO_3_H-Py), which further confirmed the synthesis of ketoenamine COFs. It was observed that the characteristic peak positions of the TpPa−(SO_3_H-Py) pattern were the same as the ^13^C patterns of TpPa−SO_3_H and TpPa−Py. Keto-enol interconversion was also observed in TpPa−(SO_3_H-Py). The peak of C=O (ketone form) was 184 ppm, and the peak of C=C (enol form) was 147 ppm and 107 ppm. The resonance peak near 134 ppm corresponded to the characteristic peak of the carbon atom connected to nitrogen in the original reactive monomer aromatic diamine. The characteristic peaks of the carbon atoms near the sulfonic acid group and the pyridine group in the TpPa−(SO_3_H-Py) skeleton were located at 125 ppm and 117 ppm. This result matched well with the ^13^C spectrum results of TpPa−(SO_3_H-Py) obtained in the previous report [24]. The above results indicated that TpPa−(SO_3_H-Py) was successfully synthesized in the SPEEK matrix.

The cross-sectional morphology of the SPEEK/TpPa−(SO_3_H-Py) composite film was observed by SEM, as shown in Figure 8d. The content of TpPa−(SO_3_H-Py) was 20 wt%, and the ratio of Pa−SO_3_H to Pa−Py was 1:1. There are no obvious cracks and defects on the cross-section of the composite membrane, and TpPa−(SO_3_H-Py) was evenly dispersed in the composite membrane.

#### 3.2.2. Dimensional Stability and Proton Conductivity

**Figure 9:**
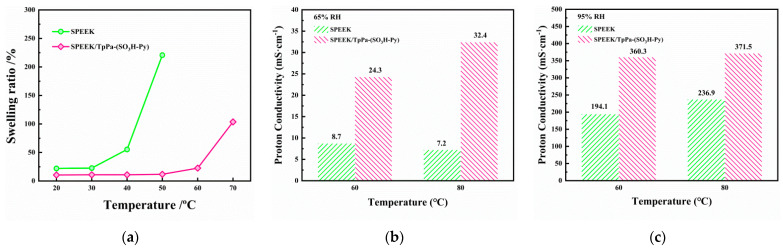
(**a**) Swelling of SPEEK and SPEEK/TpPa−(SO_3_H-Py) from 20 °C to 70 °C at 100% RH; proton conductivity at (**b**) 65%RH and (**c**) 95%RH of TpPa−(SO_3_H-Py).

The water uptaking was shown in Figure S3c and the swelling ratio of SPEEK and SPEEK/TpPa−(SO_3_H-Py) composite membranes were shown in Figure 9a. Under the condition of 20 °C and 100% RH, the water uptaking of the SPEEK and SPEEK/TpPa−(SO_3_H-Py) composite membranes were 39.3% and 44.3%, respectively; the area swelling ratios were 22.0% and 21.1%, respectively. Under 50 °C and 100% RH conditions, the water uptaking of the SPEEK and SPEEK/TpPa−(SO_3_H-Py) composite membrane were 241.5% and 56.4%, respectively. At 50 °C, the swelling ratio of SPEEK was as high as 220.7%. In contrast, the swelling ratio of SPEEK/TpPa−(SO_3_H-Py) composite membrane was only 11.8% at 50 °C, and 103.6% at 70 °C. Under high-temperature conditions, the interaction between TpPa−(SO_3_H-Py) and SPEEK limited the movement of polymer chains and improve the dimensional stability of the composite membrane. In addition, the increase in the content of pyridine groups can further enhance the interaction with SPEEK, so that the swelling ratio of the composite membrane was at a much lower level. Generally, the addition of TpPa−(SO_3_H-Py) can effectively improve the dimensional stability of the composite membrane, and the swelling inhibition effect was best when the ratio of sulfonic acid groups to pyridine groups was 1:1 (for other sulfonic acid and pyridine ratio composite membranes, please read Figure S5).

Figure 9b,c showed the conductivity of the composite membranes under different humidity environments. At 65% RH and 95% RH, the SPEEK/TpPa−(SO_3_H-Py) composite membranes showed higher proton conductivity than pure SPEEK membranes. At 60 °C and 65% RH, the proton conductivity of the SPEEK and SPEEK/TpPa−(SO_3_H-Py) composite membranes were 8.7 mS·cm^−1^, 24.3 mS·cm^−1^, respectively. When the relative humidity increased to 95%, the proton conductivity of the SPEEK and SPEEK/TpPa−(SO_3_H-Py) composite membranes were 194.1 mS·cm^−1^ and 360.3 mS·cm^−1^, respectively. At 65% RH and 95% RH, the proton conductivity of the SPEEK/TpPa−(SO_3_H-Py) composite membrane is higher than that of SPEEK membrane and SPEEK/TpPa−Py composite membranes because the −SO_3_H group constructs a hydrogen bond network and improves the proton transfer efficiency. At 80 °C and 95% RH, the conductivity of the composite membrane is as high as 371.5 mS·cm^−1^, in contrast, the conductivity of the SPEEK pristine membrane is only 236.9 mS·cm^−1^.

Compared with SPEEK/TpPa−SO_3_H composite membranes, the conductivity of SPEEK/TpPa−(SO_3_H-Py) composite membrane slightly decreased due to the reduction of −SO_3_H but showed better dimensional stability and temperature resistance. At 50 °C, the swelling ratio of SPEEK/TpPa−SO_3_H-5 composite membrane was 36.7%, while that of SPEEK/TpPa−(SO_3_H-Py) composite membrane was only 11.8%. When the temperature reached 60 °C, the SPEEK/TpPa−(SO_3_H-Py) composite membrane still maintained good dimensional stability, and the swelling rate was only 22.6%. It shows that the pyridine group significantly improves the dimensional stability of SPEEK/TpPa−(SO_3_H-Py) composite membrane. In general, the construction of TpPa−(SO_3_H-Py) with −SO_3_H group and pyridine group in the composite membranes can improve proton conductivities, dimensional stability, and temperature resistance of the composite membranes.

## 4. Conclusions

In summary, we propose a strategy for synthesizing COFs containing different groups in the SPEEK matrix to fabricate SPEEK/COFs composite proton exchange membranes. TpPa−SO_3_H containing −SO_3_H groups and TpPa−Py containing pyridine groups were synthesized in the SPEEK matrix by in situ synthesis, and evenly dispersed in the matrix. Meanwhile, the interactions between groups attached to COFs and −SO_3_H of SPEEK promote the deprotonation of –SO_3_H, constructing low-energy barrier channels for proton transport. Then the effects of different groups attached to COFs on the structure and properties of proton exchange membranes were investigated. The −SO_3_H group on the TpPa−SO_3_H framework provided additional sulfonate radicals, which enhanced the proton conductivity through the proton conduction mechanism and exhibits excellent proton conductivity. At 60 °C, under 65% RH and 95% RH, the conductivity of the SPEEK/TpPa−SO_3_H-20 composite membrane was as high as 44.1 mS·cm^−1^ and 443.6 mS·cm^−1^, which were 5.7 times and 3.3 times that of SPEEK membrane, respectively. The pyridine group on the TpPa−Py skeleton provides higher stability for the composite membrane. Benefiting from the hydrogen-bonding interfaces between the pyridine groups of TpPa−Py and SPEEK, SPEEK/TpPa−Py had good dimensional stability. The swelling ratio of SPEEK was as high as 220.7% at 50 °C, while the swelling ratio of SPEEK/TpPa−Py-25 was only 2.4%. SPEEK/TpPa−Py-20 and SPEEK/TpPa−Py-25 still maintained good dimensional stability at 80 °C, and the swelling ratios are less than 20%. Combined with the high proton conductivity of −SO_3_H and the high stability of pyridine, these two groups were introduced into the COFs skeleton at the same time. The SPEEK/TpPa−(SO_3_H-Py) composite membranes had high proton conductivity and dimensional stability. At 60 °C and 95% humidity, the conductivity of the SPEEK/TpPa−(SO_3_H-Py) composite membrane was 360.3 mS·cm^−1^, which was 1.9 times that of SPEEK, and the swelling ratio was 11.8%, which was about 1/20 of that of SPEEK. This work reveals the outstanding performance of COFs in proton conduction and the great application potential of COFs nanomaterials containing different groups in PEMs.

## Figures and Tables

**Table 1:** IEC of the membranes and DS of the SPEEK.

Membranes	IEC (mmol·g^−1^)	DS (%)
SPEEK	1.41	47.3
SPEEK/TpPa−SO_3_H-5	2.04	-
SPEEK/TpPa−SO_3_H-10	2.11	-
SPEEK/TpPa−SO_3_H-15	2.16	-
SPEEK/TpPa−SO_3_H-20	2.26	-
SPEEK/TpPa−SO_3_H-25	2.11	-
SPEEK/TpPa−Py-5	1.50	-
SPEEK/TpPa−Py-10	1.61	-
SPEEK/TpPa−Py-15	1.45	-
SPEEK/TpPa−Py-20	1.43	-
SPEEK/TpPa−Py-25	1.34	-
SPEEK/TpPa−(SO_3_H-Py)	2.09	-

## Data Availability

The data presented in this study are available on request from the corresponding author.

## Supplementary Materials

The following supporting information can be downloaded at t: https://www.mdpi.com/article/10.3390/nano12193518/s1. Table S1: Amount of raw materials required for preparing SPEEK/TpPa−SO_3_H composite membranes; Table S2: Amount of raw materials required for preparing SPEEK/TpPa−Py composite membranes; Table S3: Amount of raw materials required for preparing SPEEK/TpPa−(SO_3_H-Py) composite membranes; Figure S1: TGA of membranes; Figure S2: SEM cross-section images of composite membranes; Figure S3: Water uptaking of membranes; Figure S4: Properties of composite membranes with different ratios of sulfonic acid groups to pyridine groups; Figure S5: Proporties of composite membranes with different ratios of sulfonic acid groups to pyridine groups: (a) proton conductivity at 60 °C and 65% RH, (b) proton conductivity at 60 °C and 95% RH, (c) swelling ratio, and (d) water uptaking. Reference [37] is cited in the Supplementary Materials.
