# Supplementary material for: Effect of Covalent Organic Frameworks Containing Different Groups on Properties of Sulfonated Poly(ether ether ketone) Matrix Proton Exchange Membranes
Source: Nanomaterials (Basel). 2022 Oct 8;12(19):3518. doi: 10.3390/nano12193518 (PMC9565559; doi:10.3390/nano12193518)
Supplement: Supplementary file 1 [file nanomaterials-12-03518-s001.zip › nanomaterials-1945674-supplementary.pdf]

Effect of Covalent Organic Frameworks Containing Different Groups on Properties of Sulfonated Poly (Ether Ether Ketone) Matrix Proton Exchange Membranes

Xiaoyu Meng [1,2,*], Yinan Lv [1], Lei Ding [3], Luman Peng [1], Qiwang Peng [1], Chuanbo Cong [1,2], Haimu Ye [1,2] and Qiong Zhou [1,2]

[1] Department of Materials Science and Engineering, College of New Energy and Materials, China University of Petroleum, Beijing 102249, China
[2] Beijing Key Laboratory of Failure, Corrosion, and Protection of Oil/Gas Facilities, China University of Petroleum, Beijing 102249, China
[3] CSSC Systems Engineering Research Institute, Beijing 100036, China
* Correspondence: mengxy@cup.edu.cn

Table. S1. Amount of raw materials required for preparing SPEEK/TpPa-SO_3H composite membranes

COFs mass percentage (wt%)	Tp (g)	Pa-SO_3H (g)	PTSA (g)	20 wt% SPEEK solution (g)
5	0.0070	0.0094	0.0430	1.6380
10	0.0140	0.0188	0.0860	1.6380
15	0.0210	0.0282	0.1290	1.6380
20	0.0280	0.0376	0.1720	1.6380
25	0.0350	0.0470	0.2150	1.6380

Table. S2. Amount of raw materials required for preparing SPEEK/TpPa-Py composite membranes

COFs mass percentage (wt%)	Tp (g)	Pa-Py (g)	PTSA (g)	20 wt% SPEEK solution (g)
5	0.0092	0.0072	0.0430	1.6380
10	0.0184	0.0144	0.0860	1.6380
15	0.0276	0.0216	0.1290	1.6380
20	0.0368	0.0288	0.1720	1.6380
25	0.0460	0.0360	0.2150	1.6380

Table. S3. Amount of raw materials required for preparing SPEEK/TpPa-(SO_3H-Py) composite membranes

COFs mass percentage (wt%)	Tp (g)	Pa-SO_3H (g)	Pa-Py (g)	PTSA (g)	20 wt% SPEEK solution (g)
20	0.0316	0.0213	0.0214	0.1720	1.6380

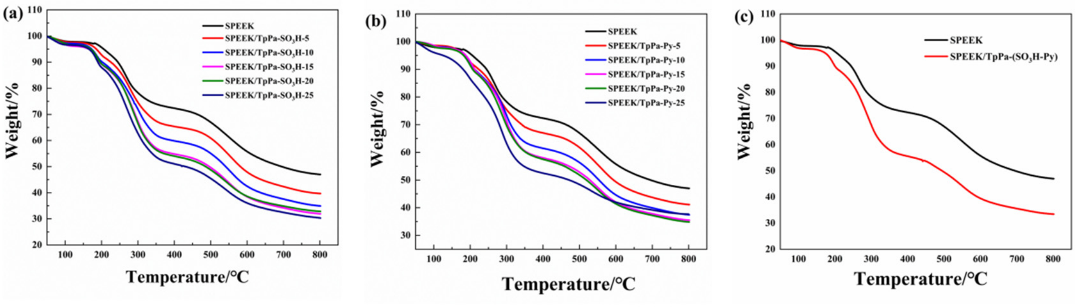

Figure S1. TGA of membranes: (a) SPEEK and SPEEK/TpPa-SO_3H, (b) SPEEK and SPEEK/TpPa-Py, (c) SPEEK and SPEEK/TpPa-(SO_3H-Py).

The thermal stability of SPEEK, SPEEK/TpPa-SO_3H, SPEEK/TpPa-Py, and SPEEK/TpPa-(SO_3H-Py) composite membranes were determined by TGA. SPEEK showed different degradation stages at 50-200℃, 200-450℃ and 450-800℃. When the temperature is in the range of 50-200℃, the mass loss of SPEEK is due to the loss of water bound by physical or chemical action. The degradation of SPEEK in the temperature range of 200-450℃ is mainly due to the decomposition of -SO_3H, and may also be accompanied by the thermal decomposition of phenol caused by random chain fracture[1]. When the temperature is above 450℃, the main chain of SPEEK breaks and pyrolysis occurs. Compared with SPEEK membranes, the mass loss of SPEEK/TpPa-SO_3H, SPEEK/TpPa-Py, and SPEEK/TpPa-(SO_3H-Py) composite membranes before 200℃ includes a large amount of proton carrier PTSA loaded in TpPa-SO_3H, TpPa-Py, and TpPa-(SO_3H-Py) channels. In the temperature range of 200-450℃, the mass loss of SPEEK/TpPa-SO_3H and SPEEK/TpPa-(SO_3H-Py) composite membrane includes not only the -SO_3H group in SPEEK matrix, but also the -SO_3H group in TpPa-SO_3H skeleton structure. The weight loss in the range of 450-800 ℃ is caused by the fracture of SPEEK polymer chain and the decomposition of COFs skeleton. Therefore, compared with SPEEK membranes, the quality of thermogravimetric curves of SPEEK/TpPa-SO_3H, SPEEK/TpPa-Py, and SPEEK/TpPa-(SO_3H-Py) composite membranes decreased significantly.

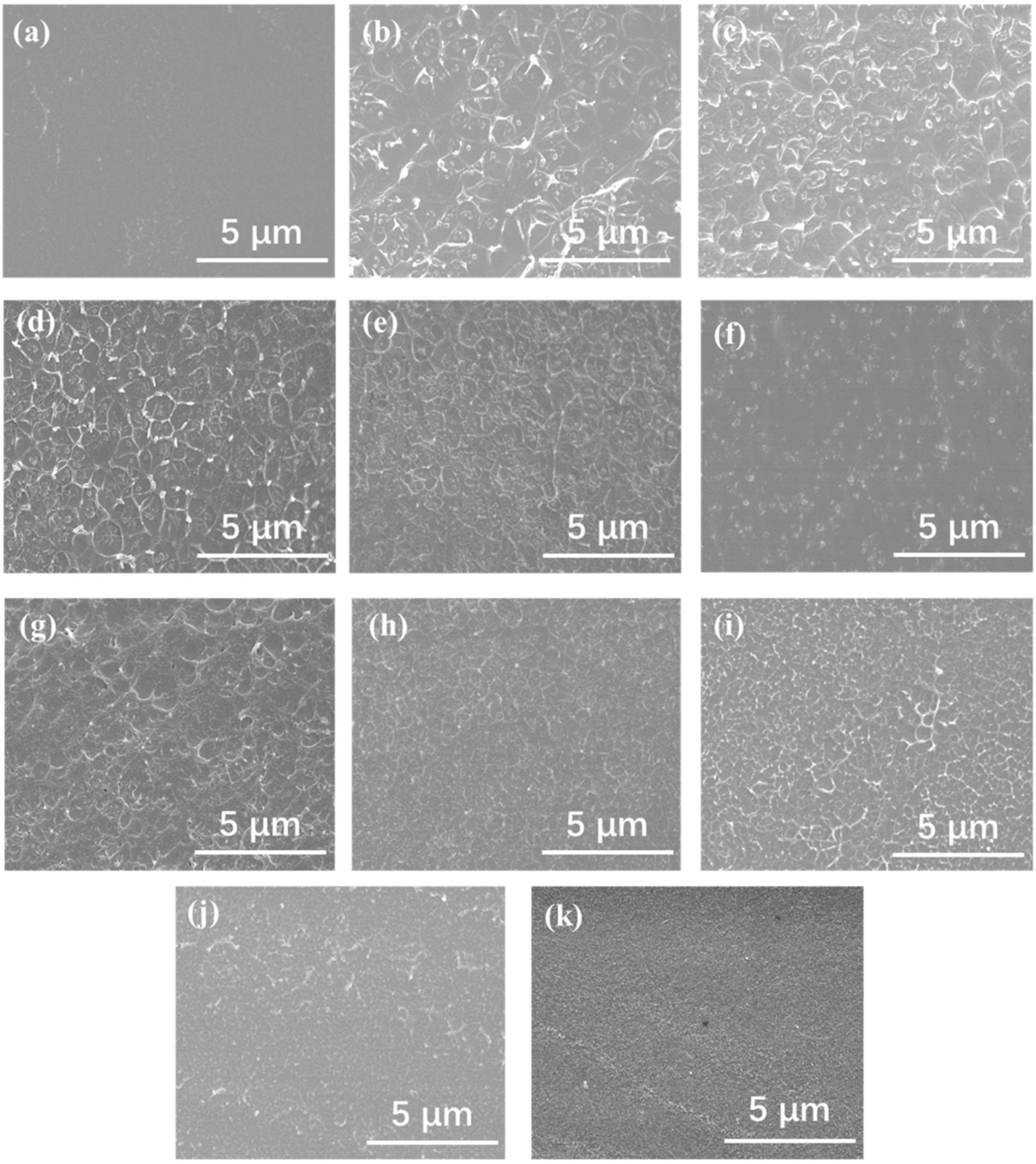

Figure S2. SEM cross-section images of (a)SPEEK, (b)SPEEK/TpPa-SO_3H-5, (c)SPEEK/TpPa-SO_3H-10, (d)SPEEK/TpPa-SO_3H-15, (e)SPEEK/TpPa-SO_3H-20, (f)SPEEK/TpPa-SO_3H-25, (g)SPEEK/TpPa-Py-5, (h)SPEEK/TpPa-Py-10, (i)SPEEK/TpPa-Py-15, (j) SPEEK/TpPa-Py-20, (k) SPEEK/TpPa-Py-25.

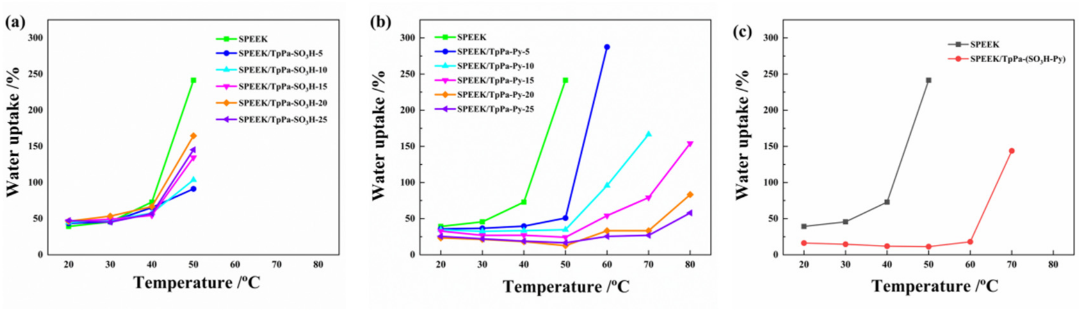

Figure S3. Water uptaking of membranes: (a) SPEEK and SPEEK/TpPa-SO_3H, (b) SPEEK and SPEEK/TpPa-Py, (c) SPEEK and SPEEK/TpPa-(SO_3H-Py).

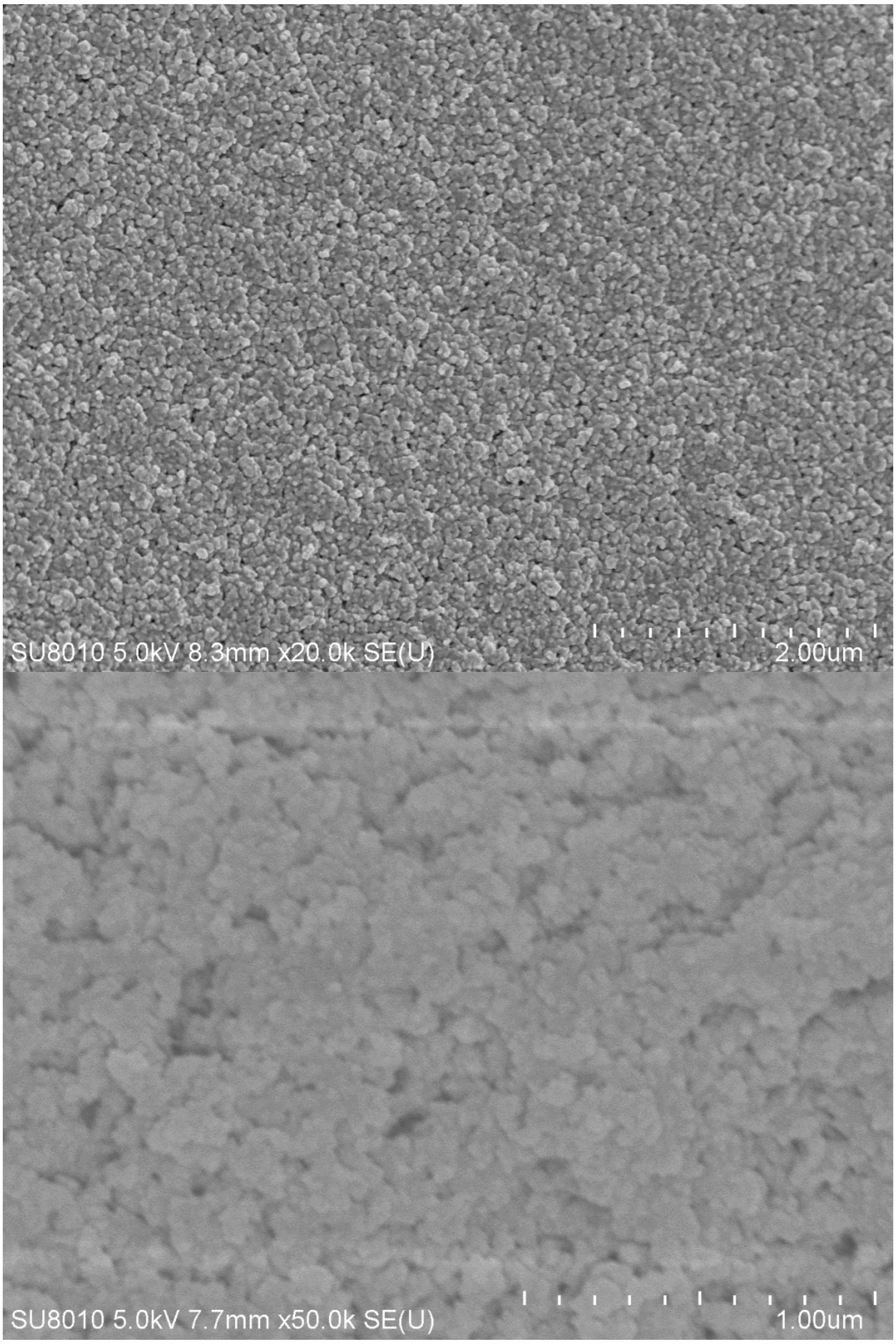

Figure S4. SEM images of TpPa-(SO_3H-Py) (×20000, and ×50000).

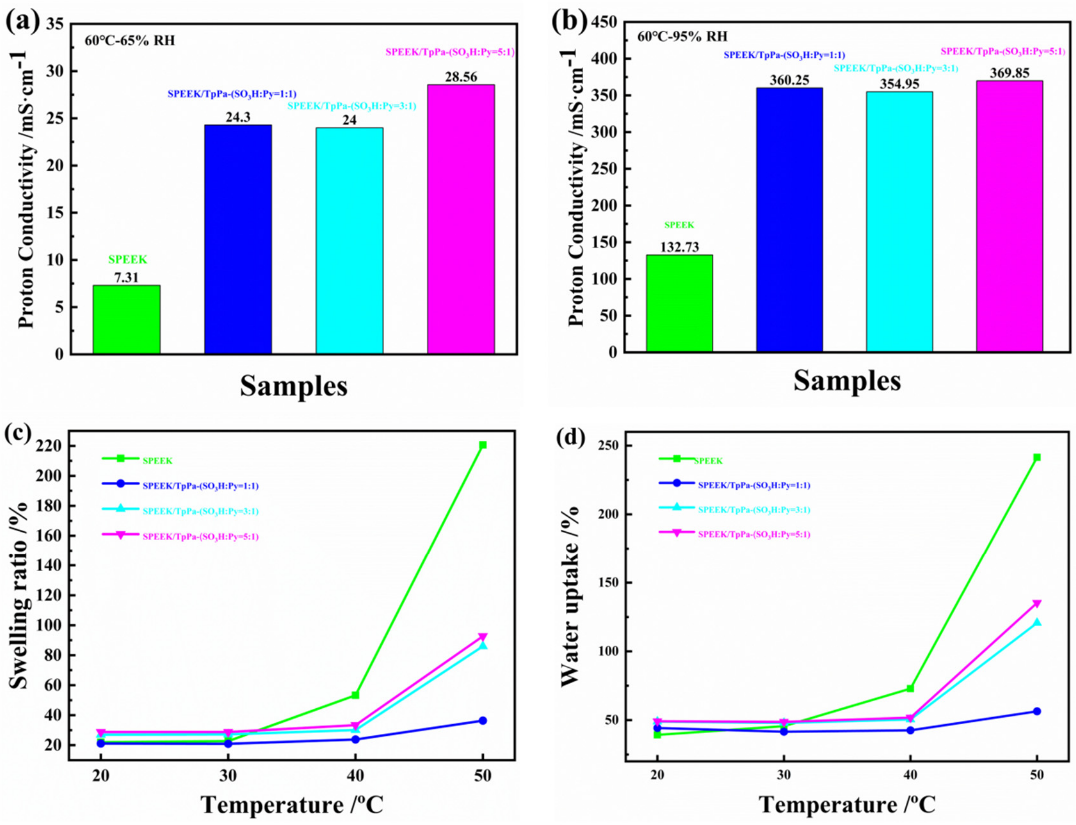

Figure S5. Proporties of composite membranes with different ratios of sulfonic acid groups to pyridine groups: (a) proton conductivity at 60°C and 65% RH, (b) proton conductivity at 60°C and 95% RH, (c) swelling ratio, and (d) water uptaking.

References

[1] Lakshmi, R. M.; Choudhary, V.; Varma, I., Sulphonated poly (ether ether ketone): Synthesis and characterisation. *Journal of materials science* **2005,** *40* (3), 629-636. https://doi.org/10.1007/s10853-005-6300-2.
